# Identification of Potential Therapeutic Targets for Coronary Atherosclerosis from an Inflammatory Perspective Through Integrated Proteomics and Single-Cell Omics

**DOI:** 10.3390/ijms26136201

**Published:** 2025-06-27

**Authors:** Hesong Wang, Fengzhe Xie, Meng Wang, Jianxin Ji, Yongzhen Song, Yanyan Dai, Liuying Wang, Zheng Kang, Lei Cao

**Affiliations:** 1 Department of Biostatistics, School of Public Health, Harbin Medical University, Harbin 150081, China; 2021020186@hrbmu.edu.cn (H.W.); 202201049@hrbmu.edu.cn (M.W.); 2020020186@hrbmu.edu.cn (J.J.); songyz1998@hrbmu.edu.cn (Y.S.); 2022020216@hrbmu.edu.cn (Y.D.); 2 Department of Social Medicine, School of Health Management, Harbin Medical University, Harbin 150081, China; xfz13030066168@163.com (F.X.); wangliuying@hrbmu.edu.cn (L.W.)

**Keywords:** coronary atherosclerosis, cardiovascular diseases, Mendelian randomization, drug target, proteomics, single-cell

## Abstract

Coronary atherosclerosis (CAS) is a major cause of cardiovascular morbidity worldwide. The understanding of atherosclerosis has shifted from a cholesterol deposition disorder to an inflammation-driven disease, with anti-inflammatory therapies demonstrating clinical efficacy. Identifying inflammatory protein targets is crucial for developing targeted therapies. A proteome-wide Mendelian randomization (MR) analysis was performed to explore therapeutic targets for CAS by integrating inflammatory proteomics data from the UK-PPP (54,219 participants, 2923 proteins) and Iceland cohorts (35,559 participants, 4907 proteins) as exposures and outcome data for CAS, atherosclerosis, and carotid atherosclerosis from FinnGen. Replication MR employed meta-analysis of six proteomics datasets and CAS data from three sources, while the impact of the identified proteins on four cardiovascular diseases was also investigated. Colocalization analysis (PPH_4_ > 0.9), reverse MR, and SMR were used to ensure robust causal inference. Proteome-wide MR identified 11 proteins significantly associated with CAS (*p* < 3.52 × 10^−5^), with all but CD4 linked to cardiovascular disease risk. Notably, colocalization confirmed the causal roles of PCSK9, IL6R, CELSR2, FN1, and SPARCL1 in CAS, and single-cell RNA-seq analysis revealed that five genes (TGFB1, SPARCL1, IL6R, FN1, and CELSR2) were exclusively expressed in smooth muscle cells of either coronary plaques or healthy vasculature. Druggability assessments were subsequently conducted for these targets. The three most promising targets (CELSR2, FN1, and SPARCL1), along with the other identified proteins and their biological functions, exhibit robust causal associations with CAS. FN1 and TGFB1 have the potential for drug repurposing in atherosclerosis treatment.

## 1. Introduction

CAS, a chronic vascular inflammatory disease, is a major contributor to cardiovascular diseases, which have become the leading cause of death worldwide [1,2]. Atherosclerotic plaques in coronary arteries can lead to severe cardiovascular events, including myocardial infarction (MI), stroke, and cardiovascular death [3,4]. Traditionally viewed as a cholesterol deposition disease, atherosclerosis is now recognized from an inflammatory perspective, which has provided more effective treatment strategies [5,6]. Clinical trials have confirmed the efficacy of anti-inflammatory approaches [7,8], yet identifying specific inflammatory therapeutic targets for atherosclerosis remains an urgent clinical need.

Proteins represent a primary source of drug targets and play critical roles in atherosclerotic disease progression. Large-scale proteomics studies have identified protein quantitative trait loci (pQTLs) [9,10,11], which represent the associations between plasma proteins and genetic variations. This provides an opportunity to identify potential drug targets for CAS from a protein-based perspective. Meanwhile, Mendelian randomization, by satisfying the three key assumptions (relevance, exchangeability, and exclusion restriction), controls for confounding and enables causal inference as an alternative to traditional RCT studies [12]. Therefore, investigating the causal relationships between specific proteins and CAS through Mendelian randomization can accelerate target drug development and deepen our understanding of atherosclerotic pathological mechanisms.

In this study, we integrated inflammation and cardiovascular-related proteins and conducted the largest study to date to identify inflammation-related protein targets for atherosclerosis. We performed Mendelian randomization using pQTL data from the UK Biobank Pharma Proteomics Project (UKB-PPP) and Iceland, as well as CAS phenotype data from the FinnGen cohort. To ensure the reliability of causal evidence, we conducted complementary analyses including colocalization analysis, reverse Mendelian randomization, and summary-data-based Mendelian randomization (SMR). Furthermore, single-cell RNA expression analysis was used to investigate the differences in expression patterns of the identified causal proteins between atherosclerotic and healthy vessels. Additionally, the associations of these proteins with cardiovascular diseases were also investigated.

## 2. Results

### 2.1. Proteome Screening for Causal Proteins in CAS

**Figure 1:**
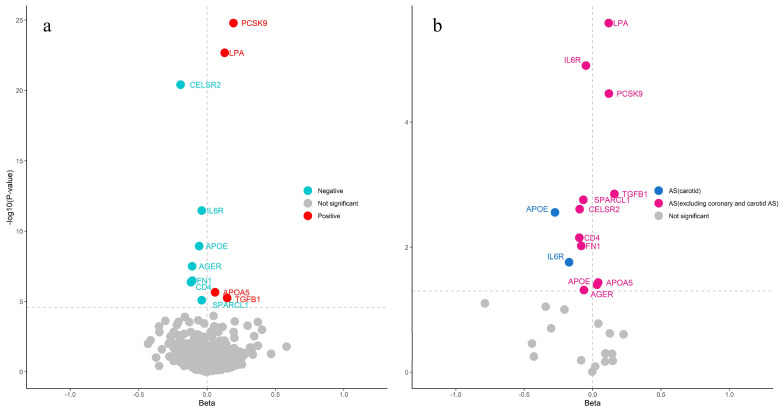
The volcano plot illustrates the MR analysis results between proteins and atherosclerosis. (**a**) The 11 identified proteins in CAS (Horizontal dotted line: *p* < 3.52 × 10^−5^). (**b**) Significantly identified proteins in atherosclerosis and carotid atherosclerosis (Horizontal dotted line: *p* < 0.05).

Following lead cis-pQTL and multi-instrument proteomic MR analyses, 11 causal proteins (seven in the inflammation category and four in the cardiovascular category) were ultimately identified as being associated with CAS risk at the Bonferroni significance level (*p* < 3.52 × 10^−5^) (Figure 1a and Table 1). Instrumental variables were selected based on the above criteria to satisfy the three MR assumptions. No heterogeneity was detected among all the proteins. The expression of four proteins (PCSK9, LPA, APOA5, TGFB1) was associated with an increased risk of CAS. The expression of the remaining seven (CELSR2, APOE, IL6R, FN1, AGER, CD4, SPARCL1) was associated with a decreased risk of CAS. All proteins passed the heterogeneity test (*p* > 0.05). SMR analysis was also applied to the identified proteins, and all proteins showed a significant association with CAS. These identified proteins can be considered highly reliable. The discovery MR and SMR results are presented in Supplementary Tables S5–S7.

In other types of atherosclerosis, all 11 causal proteins (PCSK9, LPA, IL6R, TGFB1, SPARCL1, CELSR2, CD4, FN1, APOA5, APOE, and AGER) were found to be associated with the risk of atherosclerosis, excluding coronary and carotid atherosclerosis, and two proteins (IL6R, APOE) were associated with the risk of carotid atherosclerosis (Figure 1b). This finding demonstrates that CAS shares causal proteins with atherosclerosis but not with carotid atherosclerosis, which aligns with the fact that carotid atherosclerosis is generally linked to cerebrovascular diseases.

### 2.2. Causal Proteins for Cardiovascular Disease

**Figure 2:**
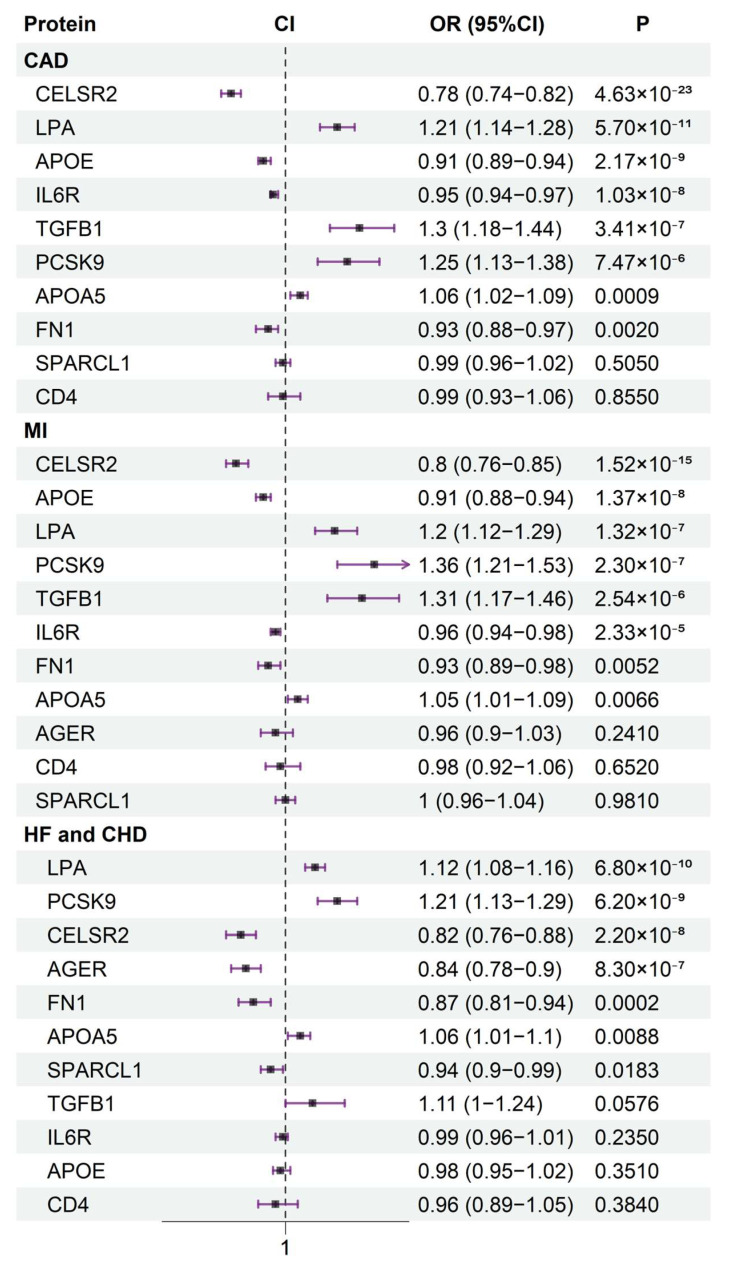
The association between 11 identified proteins and cardiovascular diseases (CAD, MI, HF, and CHD) risk.

To investigate the effects of the identified proteins on cardiovascular disease, we conducted MR analysis using the 11 causal proteins as exposures, with CAD and myocardial infarction (MI) data from the CARDIoGRAMplusC4D Consortium as well as heart failure (HF) and coronary heart disease (CHD) data from the Fenland study as outcomes. Ultimately, except for CD4, all other proteins were associated with the risk of at least two cardiovascular diseases (Figure 2). This further indicates the potential impact of the identified proteins on atherosclerosis, as it is closely linked to the progression of cardiovascular diseases.

### 2.3. Bayesian Colocalization and Reverse MR for the Causal Relationship Between Causal Proteins and CAS

Bayesian colocalization and reverse MR were used to exclude causal pathways other than from causal proteins to CAS. PCSK9 (rs11591147, PPH_4_ = 0.999), CELSR2 (rs12740374, PPH_4_ = 0.997), IL6R (rs4129267, PPH_4_ = 0.986), FN1 (rs1250259, PPH_4_ = 0.972), CD4 (rs73053728, PPH_4_ = 0.983), and SPARCL1 (rs7681694, PPH_4_ = 0.963) share the same variant loci with CAS (Table 1 and Supplementary Table S9). The LocusZoom plots illustrating the cis regions of all candidate protein targets and the corresponding CAS-related regions are presented in Supplementary Figures S1–S11. Linkage disequilibrium and pleiotropy were excluded through colocalization analysis. Additionally, the PPH_3_ of APOE, LPA, APOA5, AGER, and TGFB1 was greater than 0.9, indicating that these proteins share non-independent genetic variants with CAS. This does not rule out the causal relationship between the proteins and CAS, and further research is needed to explore the underlying mechanisms. The reverse MR results for all identified proteins were consistent, indicating that the causal direction is from protein to CAS (Table 1 and Supplementary Table S8). It is worth noting that the reverse MR for CELSR2 was significant (*p* < 0.05), but it did not pass the heterogeneity test, so we conclude that it does not have a reverse causal relationship with CAS.

### 2.4. Replication Validation for Identified Proteins

**Figure 3:**
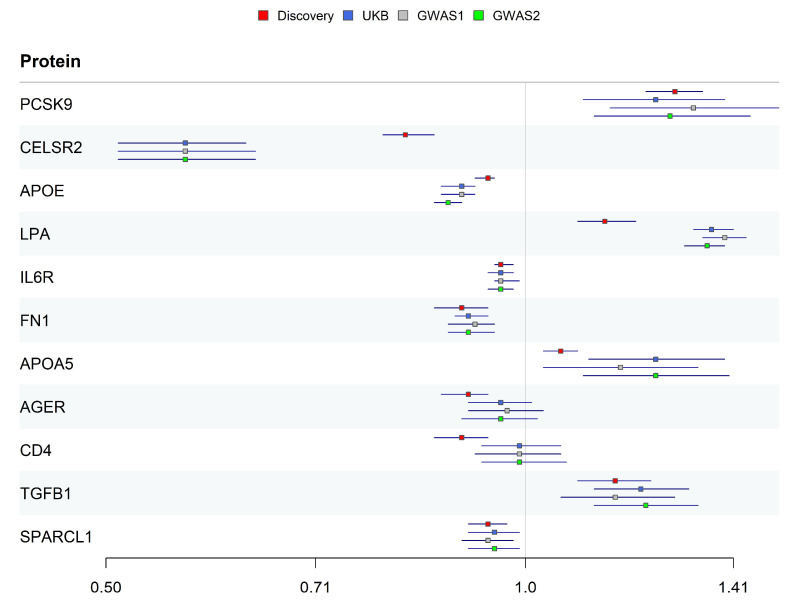
OR estimates of 11 candidate proteins in discovery and replication datasets.

Replication MR was performed three times to ensure the robustness of causal relationships using integrated pQTL data (five pQTL studies and Norfolk cohort) as exposure and three CAS phenotype datasets (UK Biobank and GWAS Catalog). Only CD4 and AGER did not pass the replication validation at the significance level of 0.05 (Figure 3).

The identified causal proteins were classified into three tiers. Five proteins (PCSK9, CELSR2, IL6R, FN1, and SPARCL1) passed all validations and were classified as Tier 1. Three proteins (APOE, LPA, and TGFB1) did not pass colocalization validation and were classified as Tier 2. Two proteins (AGER and CD4) did not pass replication validation and were classified as Tier 3 (Table 1).

### 2.5. Single-Cell RNA-Seq Differential Expression Analysis for Coronary Plaque Samples and Healthy Vasculature

**Figure 4:**
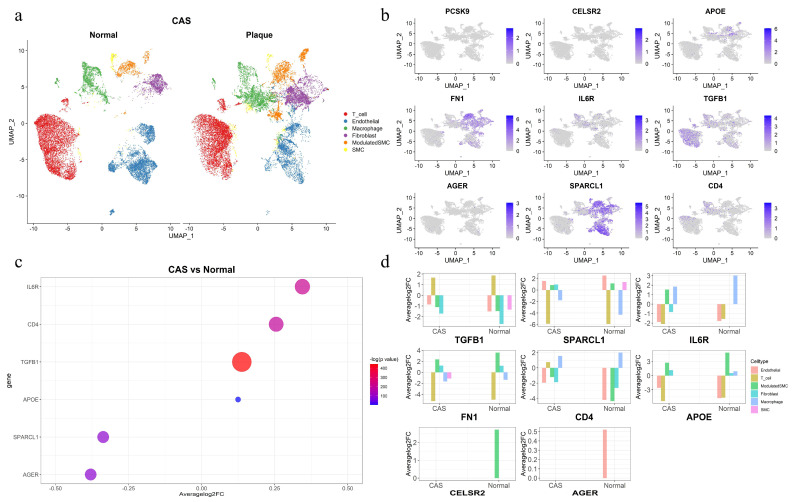
Single-cell expression of the identified candidate protein-coding genes in coronary plaques and healthy vasculature. (**a**) Six cell types were clustered between coronary plaques and healthy vasculature. (**b**) The expression of nine protein-coding genes in coronary plaques. (**c**) Six differentially expressed genes between coronary plaques and healthy vasculature. (**d**) The differential expression of eight protein-coding genes across different cell types in coronary plaques and healthy vasculature compared to other cell types.

To investigate the expression of the 11 identified protein-coding genes in the coronary arteries and their expression patterns across different cell types, single-cell RNA-seq differential expression analysis was performed in coronary plaques and healthy vasculature. Cells were clustered into 15 clusters and ultimately classified into six cell types (T cell, endothelial, macrophage, fibroblast, modulated SMC, smooth muscle cell) (Figure 4a). Nine of the eleven proteins were expressed in coronary plaques and healthy vasculature, while *APOA5* and *LPA* were not detected (Figure 4b). *FN1*, *SPARCL1*, *IL6R*, *TGFB1*, and *APOE* were enriched in immune cells and SMC. Immune dysregulation within the plaque microenvironment of CAS may contribute to clinical ischemic stroke or myocardial infarction [13], while SMCs represent a predominant cell type throughout all stages of atherosclerosis, exhibiting significant heterogeneity in both morphology and atherosclerosis-related gene expression [5]. We identified six genes (*IL6R*, *CD4*, *TGFB1*, *APOE*, *SPARCL1*, *AGER*) with differential expression in coronary plaques and healthy vasculature at a log2FC > 0.1 level. (Figure 4c) The same differential expression analysis was performed across different cell types. Eight of the nine genes exhibited differential expression across cell types, with only PCSK9 showing no differential expression (Figure 4d). Smooth muscle cells (SMCs) are a key cell type in the pathological process of atherosclerosis. *TGFB1*, *SPARCL1*, *IL6R*, *FN1*, and *CELSR2* exhibited differential expression in smooth muscle cells (SMCs), which was observed as differential expression only in the SMCs of the plaque group or healthy vasculature group compared to other cell types. Summary statistics of the differential expression of protein-coding genes across various cell types are provided in Supplementary Table S13.

### 2.6. Protein–Protein Interaction (PPI) Network, Enrichment Analysis, and Drug Repurposing

**Figure 5:**
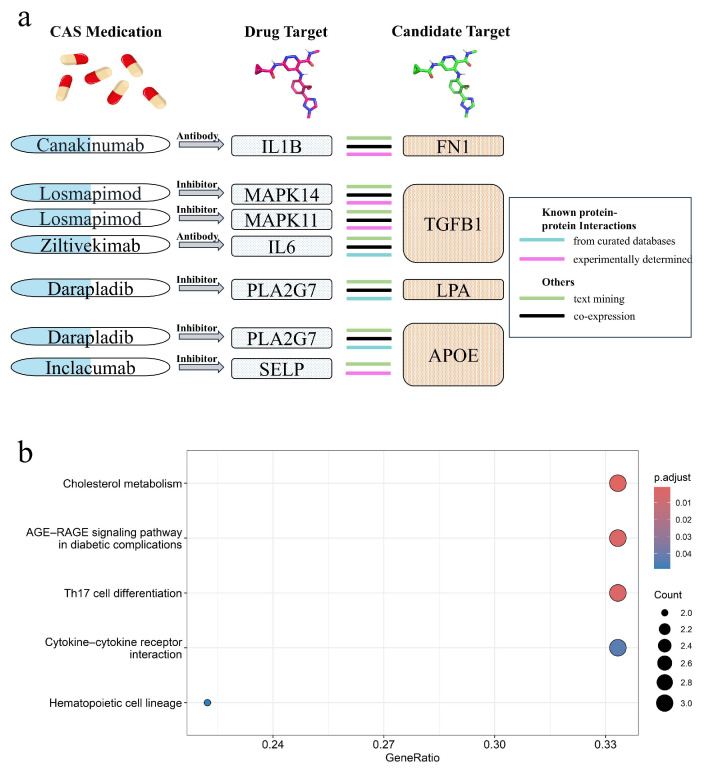
Protein–protein interaction (PPI) analysis and pathway enrichment analysis. (**a**) Interactions between CAS drug targets and identified potential drug targets (FN1, TGFB1, LPA, APOE). (**b**) Top five enriched pathways of the identified proteins.

PPI analysis was conducted to investigate the association between the identified proteins and current atherosclerosis inflammation drug targets in clinical trials (Supplementary Figure S12). Among the 11 identified proteins, all except SPARCL1 interact with current CAS drug targets in clinical trials (IL6R and PCSK9 are established CAS drug targets). FN1, TGFB1, LPA, and APOE exhibit highly credible interactions with CAS drug targets (Figure 5a). Gene Ontology (GO) enrichment analysis revealed that these proteins are primarily associated with the regulation of the ERK1 and ERK2 cascade, which is dysregulated in diabetes and inflammation [14], as well as lipid and cholesterol metabolism. Enrichment analysis also showed that these proteins are enriched in cholesterol metabolism, the AGE-RAGE signaling pathway, and cytokine–cytokine receptor interaction (Figure 5b). The binding of AGE to RAGE induces multiple signaling pathways, leading to inflammation, atherosclerosis, and vascular constriction [15]. Along with the role of cytokine–cytokine receptor interaction in immune regulation and inflammatory responses [16], and the impact of cholesterol metabolism on atherosclerosis [17], these findings highlight the potential role of these proteins in CAS.

Among the 11 candidate proteins, PCSK9 (Alirocumab, approved) and IL6R (Tocilizumab, Phase 3) are current drug targets for atherosclerosis in clinical trials. Ocriplasmin (Approved), which targets FN1 and is used to treat vitreomacular adhesion, and two drugs targeting TGFB1, foreskin keratinocyte (Approved), a treatment used to accelerate wound healing, and terazosin (Approved), a medication for benign prostatic hyperplasia and hypertension, may have potential for repurposing in the treatment of atherosclerosis and cardiovascular diseases. However, clinical evidence confirming its pharmacological effects is currently lacking, and further clinical studies are needed to support this potential application. The detailed drug information and sources for the candidate protein targets are provided in Supplementary Table S12.

## 3. Discussion

Coronary atherosclerosis is closely linked to the progression of cardiovascular diseases. This study aims to identify therapeutic targets for atherosclerosis from an inflammation perspective. To our knowledge, this is the largest study to date focusing on inflammation-related protein targets for coronary atherosclerosis, incorporating the most rigorous replication validation across four independent datasets.

Through proteome-wide Mendelian randomization analysis, 11 proteins (seven inflammation-related and four cardiovascular-related) were identified to be associated with CAS risk at a significance threshold of *p* < 3.52 × 10^−5^, some of which were also linked to the development of atherosclerosis and carotid atherosclerosis. After rigorous exclusion of alternative causal pathways, replication validation, and colocalization analysis, five proteins (PCSK9, CELSR2, IL6R, FN1, SPARCL1) were classified as tier 1, supported by the highest level of evidence. Four proteins (APOE, LPA, APOA5, TGFB1) that did not pass colocalization analysis were classified as tier 2, supported by moderate evidence. Two proteins (AGER, CD4) that failed replication validation were classified as tier 3. We also investigated the impact of the identified proteins on cardiovascular diseases (CAD, MI, HF, and CHD). Except for CD4, all proteins were associated with cardiovascular disease risk. Considering the function of CD4 and its lack of association with cardiovascular diseases in our analysis, it is not regarded as a potential drug target. Additionally, single-cell RNA-seq data were utilized to analyze the expression patterns of these candidate protein-coding genes across different cell types in healthy vasculature and coronary plaques. PCSK9 and IL6R are current therapeutic targets under investigation for atherosclerosis. Additionally, one existing drug targeting FN1 and two drugs targeting TGFB1 have the potential for repurposing in atherosclerosis treatment.

Cadherin EGF LAG seven-pass G-type receptor 2 (CELSR2) is a member of the Flamingo subfamily, which belongs to the cadherin superfamily. Although the underlying mechanism remains unclear, previous GWASs have identified an association between CELSR2 and coronary artery disease as well as serum cholesterol metabolism [18,19]. Additionally, CELSR2 negatively regulates JNK signaling, which affects the Wnt/β-catenin pathway [20], and the Wnt pathway is directly linked to VSMC proliferation in neointima formation during atherosclerosis [21]. Moreover, SMCs play a crucial role in all stages of atherosclerotic plaque development [22], which is consistent with our finding that CELSR2 exhibits differential expression in modulated SMCs between healthy vasculature and plaque samples. We also identified the protective effect of CELSR2 on cardiovascular diseases (CAD, MI, HF, CHD), suggesting its potential as a therapeutic target for CAS.

Fibronectin (FN) is an extracellular matrix (ECM) protein. It is a key player in vascular biology, contributing to embryonic development, various cardiovascular diseases, and pathological processes where vascular development is critical, such as tumor progression [23]. In atherosclerosis, FN is believed to exacerbate disease progression by mediating inflammatory effects through integrin receptors (e.g., α5β1) [24]. Previous studies have linked elevated plasma FN (pFN) levels to atherosclerosis and ischemic heart disease [25,26]. Rohwedder et al. reported that pFN may promote plaque rupture and arterial occlusion by regulating FN production, while vascular FN deposits serve as a trigger for vascular SMC recruitment, strongly opposing the therapeutic strategy of blocking FN function to treat atherosclerosis [27]. Our study found that FN expression in SMCs within coronary plaques was lower than in other cell types and exhibited a protective effect against CAS (OR = 0.9 [0.86–0.94]), supporting the findings of Rohwedder et al. Doddapattar et al. [28,29] demonstrated that the addition of human cellular fibronectin (cFN, containing EDA, 0–50 μg/mL) to bone marrow-derived macrophages dose-dependently activated NF-κB p65 and linearly upregulated TNFα and IL-1β expression. At concentrations of 10 μg/mL and 50 μg/mL, MMP-9 expression was significantly elevated, suggesting that high-dose FN promotes inflammation and matrix degradation, which may exacerbate atherosclerosis and increase the risk of plaque instability in advanced stages. However, earlier studies, including mouse knockout experiments [27] and pathological comparative research [30], have shown that FN1 suppresses pro-inflammatory signaling, thereby reducing both the number and size of atherosclerotic plaques. These findings suggest that fibronectin may act as a double-edged sword. Moderate upregulation of FN could serve as a potential therapeutic strategy for atherosclerosis, although further research is needed to clarify the underlying mechanisms.

SPARC-like protein 1 (SPARCL1) is an extracellular matrix protein involved in regulating cell adhesion, proliferation, and migration; promoting cellular quiescence and vascular endothelial homeostasis [31]; and exhibiting tumor-suppressive effects in certain cancers [32,33]. Endothelial dysfunction is a critical initiating factor in atherosclerosis, and the role of SPARCL1 in stabilizing the vascular endothelium [34], along with its confirmed involvement in atherosclerosis development [35], suggests its potential as a therapeutic target for the disease. SPARCL1 exhibited differential expression between coronary plaques and healthy vasculature in our single-cell analysis and was also negatively associated with the occurrence of HF and CHD. Epidemiological studies [36] have shown that elevated serum SPARCL1 levels are associated with lower triglyceride levels, increased HDL-C, and a reduced risk of dyslipidemia. Specifically, each one standard deviation increase in SPARCL1 was associated with approximately 20% and 12% lower risks of hypertriglyceridemia and overall dyslipidemia, respectively. SPARCL1 has been widely studied in the context of cancer progression, and knockout of the Sparcl1 gene in mice has been shown to significantly increase vascular permeability [37]. Transcriptomic analyses suggest that Sparcl1 may contribute to the maintenance of vascular wall stability [31]. Notably, SPARCL1 expression has been confirmed in atherosclerotic tissues [35]; however, whether it plays a protective or promotive role in atherosclerosis remains unclear. Our findings indicate a potential protective role of SPARCL1 in CAS, highlighting the need for future studies to further investigate this effect.

Among the candidate target proteins outside Tier 1, APOE, LPA, and APOA5, as members of the apolipoprotein family, are involved in lipid and cholesterol metabolism. Substantial evidence has long established the association between APOE and coronary artery disease [38] as well as the severe hyperlipidemia and atherosclerosis observed in *ApoE*^−/−^ mice [39]. Lipoprotein (a) (LPA) is a low-density lipoprotein-like particle with extensive preclinical evidence supporting its association with CAD [40]. Currently, no approved therapies targeting LPA for CAD exist, but LPA is associated with an increased risk of cardiovascular diseases (CAD, MI, HF, CHD). LPA-lowering therapies may hold potential for atherosclerosis treatment. Currently, no approved drugs target APOA5 for the treatment of coronary artery disease, and previous epidemiological studies and fundamental experiments have also indicated its association with CAD [41].

Studies investigating the relationship between transforming growth factor beta-1 proprotein (TGFB1) and cardiovascular diseases are relatively limited. TGFB1 can enhance the phosphorylation of Smad2 and Smad3, promoting the formation of a heterotrimeric complex with Smad4, thereby exacerbating vascular fibrosis and stiffness [42]. Moreover, TGFB1 interacts with TNFSF10 to activate the TGFβ and NF-κB pro-inflammatory pathways, inducing the paradox of carotid atherosclerotic calcification [43]. However, these mechanisms require further investigation. Currently, AGEs have been found to be associated with cardiovascular diseases and diabetic complications [44].

The strength of this study lies in the rigorous exclusion of alternative causal pathways, three independent replication analyses, and further exploration of their associations with cardiovascular diseases (CAD, MI, HF, and CHD). Combined with relevant biological evidence, this enhances the potential of these targets as therapeutic options for CAS. Furthermore, we exclusively utilized cis-pQTLs to ensure that genetic variants serve as reliable proxies for protein levels and to enhance biological interpretability. Additionally, single-cell analysis between coronary plaques and healthy vasculature provides further support for mechanistic exploration. To our knowledge, this is the largest study to date exploring atherosclerosis targets from an inflammation perspective, identifying nine potential therapeutic targets for CAS, excluding CD4 and AGER (lacking colocalization and replication evidence). Among them, PCSK9 and IL6R are already being investigated as therapeutic targets for atherosclerosis. CELSR2, FN1, SPARCL1, APOE, LPA, APOA5, and TGFB1 exhibit causal associations with both atherosclerosis and four cardiovascular diseases, suggesting their potential as therapeutic targets for atherosclerosis. Notably, FN1 and TGFB1 have approved drugs targeting other diseases, which may be repurposed for atherosclerosis treatment. However, this therapeutic potential requires further clinical trials to validate their efficacy in this context. This study also has certain limitations. First, the stringent significance threshold for identifying proteins may have excluded some potential proteins that could influence the progression of CAS. Moreover, the study population primarily consisted of individuals of European ancestry, which may limit its generalizability to non-European populations. Finally, as CD4 and AGER lacked replication evidence, their association with CAS should be interpreted with caution.

## 4. Materials and Methods

### 4.1. Study Design

**Figure 6:**
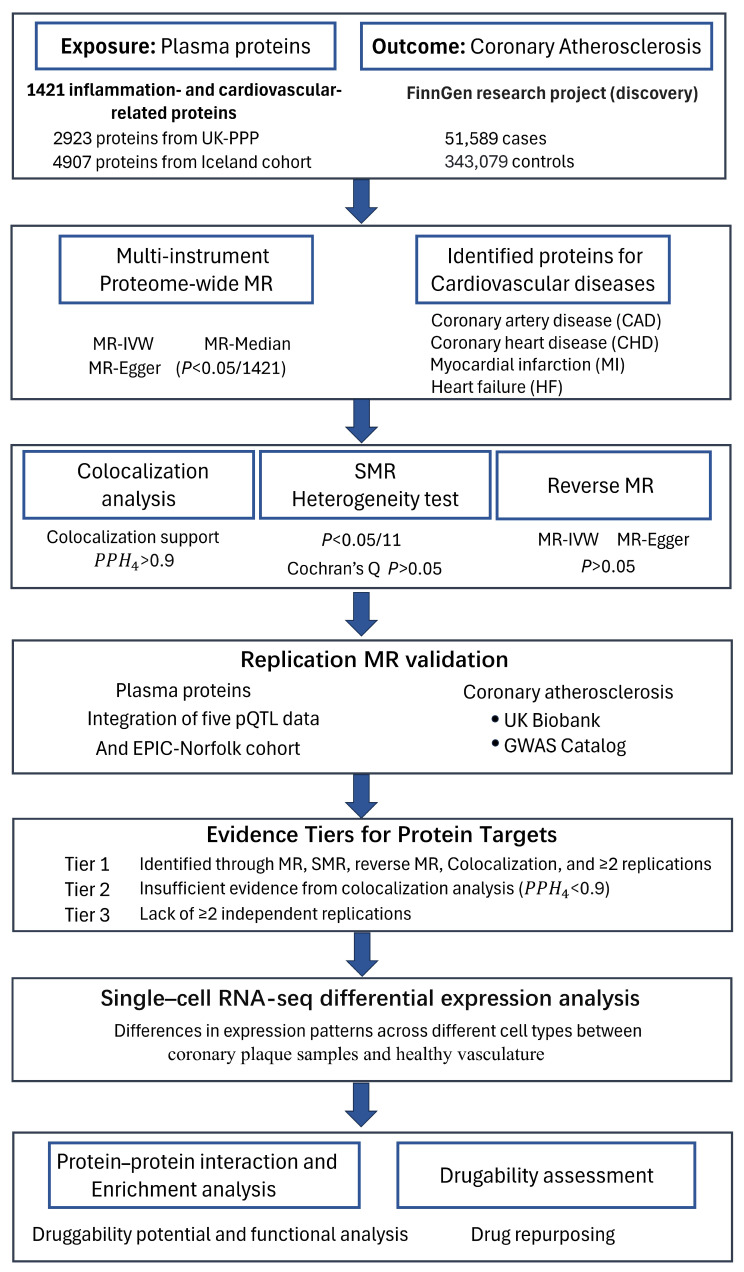
Study design for identifying causal associations between inflammatory plasma proteins and CAS.

The design of this study is presented in Figure 6 and strictly follows the STROBE-MR (strengthening the reporting of observational studies in epidemiology using Mendelian randomization) [45]. We primarily used MR (relevance, exchangeability, and exclusion restriction), fulfilling all three assumptions, to explore the causal relationship between inflammatory proteins and CAS [12]. First, we employed Mendelian randomization (MR) using the lead cis-pQTLs of 1421 inflammation- and cardiovascular-related proteins as instrumental variables to identify significant proteins for CAS, with Bonferroni-corrected *p*-values (0.05/1421). To enhance the reliability of our findings, multi-instrument Mendelian randomization (MR) was applied to the identified significant proteins for CAS (*p* < 0.05/1421). The associations between these identified proteins and cardiovascular disease risk were also explored. Next, SMR further ensured the robustness of the results. Bayesian colocalization and reverse Mendelian randomization were employed to eliminate the possibility of other causal relationships. Reproducibility of the results was demonstrated through multiple rounds of Mendelian randomization. Then, single-cell expression analysis between coronary plaque samples and healthy vasculature was performed to identify differential expression patterns of the identified causal proteins across different cell types. Finally, protein–protein interaction (PPI) analysis and enrichment analysis were used to evaluate the druggability. The repurposing potential of existing drugs for atherosclerosis was also evaluated. To prioritize candidate protein targets, we classified the evidence into three tiers. Proteins that passed all evaluations, including MR (*p* < 0.05/1421), SMR (*p* < 0.05/11), reverse MR (*p* > 0.05), Bayesian colocalization (PPH_4_ > 0.9), and at least two independent replications, were designated as Tier 1. Proteins that did not pass the colocalization analysis were classified as Tier 2. Proteins lacking at least two independent replications were assigned to Tier 3.

### 4.2. Proteomic Data Source

Summary GWAS (pQTL) data on plasma protein levels were obtained in the discovery phase from two large-scale proteomics projects: the UK Biobank Pharma Proteomics Project [46] (54,219 UK Biobank participants, 2923 proteins) and the Iceland cohort [9] (35,559 Icelanders, 4907 proteins). A total of 7830 inflammation- and cardiovascular-related plasma proteins were integrated from these two projects. The classification of plasma protein associations was determined based on the panels of two proteomics platforms, SomaScan version 4 [47] and Olink Explore 3072/384 [48]. In the replication phase, pQTL data were obtained from a meta-analysis of five GWAS [49] and the EPIC-Norfolk cohort [50]. The detailed information of plasma protein data is provided in Supplementary Table S1. Protein levels measured using the two platforms, SomaScan and Olink, were independently normalized within each dataset. pQTL analyses were then conducted in comparable populations, with appropriate adjustment for baseline covariates. In addition, a cross-platform comparison by Sun et al. [46] demonstrated that 81% of the primary associations identified using the Olink platform were replicated across 34 independent studies. The use of lead cis pQTLs further minimized heterogeneity in the integrated analysis.

### 4.3. GWAS Summary Statistics for Atherosclerosis and Cardiovascular Diseases

GWAS data for CAS ($n_{case}$ = 51,589, $n_{control}$ = 343,079, prevalence = 13.07%), atherosclerosis (excluding cerebral, coronary, and PAD) ($n_{case}$ = 16,243, $n_{control}$ = 381,977, prevalence = 4.08%), and carotid atherosclerosis ($n_{case}$ = 342, $n_{control}$ = 411,839, prevalence = 0.83‰) in the discovery phase were obtained from the FinnGen research project [51]. CAS data from Pan UKBB [52] ($n_{case}$ = 23,888, $n_{control}$ = 382,052, prevalence = 5.88%) and the GWAS Catalog [53,54] were used for replication validation.

We primarily explored the relationship between the risk of four cardiovascular diseases (CAD, MI, HF, and CHD) and the identified proteins. The CAD and MI phenotype data were obtained from the CARDIoGRAMplusC4D Consortium [55,56]. The GWAS data for HF and CHD were all obtained from the FinnGen research project. The detailed information of atherosclerosis and cardiovascular diseases phenotype data is provided in Supplementary Table S2.

### 4.4. Proteome-Wide MR Analysis for Identifying Causal Proteins

Mendelian randomization (MR) was performed using the R (V4.3.3) package “MendelianRandomization” (V0.10.0) to identify causal inflammatory proteins for CAS. The protein pQTL data and GWAS phenotype data are derived from two sources with no overlap. The selection of instrumental variables was based on the following four criteria: (i) a significant Bonferroni-corrected genetic association threshold (5 × 10^−8^) to control type I errors; (ii) the pQTL must be a cis-pQTL, with the genetic association located within ±500 kb of the protein-coding gene region to eliminate pleiotropy; (iii) linkage disequilibrium (LD) clumping (r^2^ < 0.001) was used to ensure the independence of genetic associations and remove horizontal pleiotropy [49]; (iv) the instrument strength (F-statistic) must be greater than 10 to ensure strong correlation [57].

We first performed single-instrument MR using lead pQTLs (lowest *p*-value associations) as instrumental variables for exploratory analysis. Then, we conducted multi-instrument MR analysis on the identified proteins with multiple corrections (*p* < 3.52 × 10^−5^, 0.05/1421). Finally, SMR analysis was performed using the “gsmr” (V1.0.6) R package to ensure the robustness of the results [58]. In the replication phase, MR was performed on the identified inflammatory proteins using different plasma proteins and CAS datasets, with a significance threshold of *p* < 0.05. The inverse variance-weighted (IVW) method was used to estimate the effect size (ratio estimate for a single instrument), and heterogeneity tests were conducted to exclude instrumental variable heterogeneity (Cochran’s Q *p* > 0.05). Instrument variables for lead cis-pQTL MR and multiple cis-pQTLs MR are presented in Supplementary Tables S3 and S4.

To eliminate the possibility of reverse causality for the identified proteins, reverse MR was performed using the MR-IVW and MR-Egger methods (*p* > 0.05). For other types of atherosclerosis, we also performed multi-instrument MR using the identified inflammatory proteins as exposures and atherosclerosis and carotid atherosclerosis as outcomes.

### 4.5. Bayesian Colocalization Analysis

Bayesian colocalization analysis was performed to assess whether inflammatory proteins and atherosclerosis share the same genetic variant, providing further support for causal evidence [59]. The colocalization region was defined as ±500 kb around the protein-coding gene region and the corresponding gene region for CAS. Colocalization analysis considers five hypotheses: H_0_, where no variants are associated with either the protein or CAS; H_1_, where genetic variants are associated only with the protein; H_2_, where genetic variants are associated only with CAS; H_3_, where two independent variants are associated with the protein and CAS; and H_4_, where a shared variant is associated with both the protein and CAS. A posterior probability of H_4_ (PPH_4_ > 0.9) was considered evidence of a causal relationship between the identified protein and CAS, indicating that both the protein and the disease are driven by a shared genetic variant [59]. The “coloc” (V5.2.3) R package was used to perform colocalization analysis, and the results were visualized using the “locuszoomr” (V0.3.8) R package [60].

### 4.6. Single-Cell RNA-Seq Differential Expression Analysis

Single-cell RNA-seq gene differential expression analysis was performed to identify the expression patterns of the identified protein-coding genes between coronary plaque samples and healthy vasculature. Human CAS plaque samples and adaptive intimal thickening (AIT, representing healthy vasculature) samples were obtained from the GEO database (GSE252243) [61]. Sc RNA-seq data processing and analysis were implemented using the R package “Seurat” (V5.2.1) [62]. During the quality control phase, we excluded genes with fewer than three cell counts and cells with fewer than 200 feature counts. A total of 36,940 genes were retained, with 18,558 cells in the plaque group and 15,648 cells in the healthy vasculature group. Marker genes for six cell types were used for cell annotation (Supplementary Table S10). After normalization, we integrated cells using the Harmony method and performed differential expression analysis between CAS plaque samples and healthy vasculature samples, as well as between cell types, using the Wilcoxon Rank Sum test. A log fold change > 0.5 and a multiple correction-adjusted *p*-value < 0.05 were considered statistically significant.

### 4.7. Protein–Protein Interaction, Enrichment Analysis, and Druggability Assessment

To investigate the association between the identified protein targets and inflammatory drug targets for atherosclerosis, we performed a protein–protein interaction analysis using the STRING (V12.0) database [63]. Inflammatory target proteins were obtained from Kong et al. [5], who compiled current clinical trial drugs for atherosclerosis, with detailed drug information sourced from the ChEMBL [64] and DrugBank [65] databases (Supplementary Table S11). GO term and pathway enrichment analyses were performed using the “clusterProfiler” R package [66] to explore the biological functions of the identified proteins. Additionally, we evaluated the potential repurposing of existing drugs for CAS.

## 5. Conclusions

The three most promising proteins (CELSR2, FN1, and SPARCL1) were identified as potential therapeutic targets for atherosclerosis, while APOE, LPA, APOA5, TGFB1, and AGER also hold potential as drug targets for the disease. All identified proteins are supported by robust causal associations; however, further research, particularly clinical studies, is needed to elucidate their underlying mechanisms.

## Figures and Tables

**Table 1:** Summary of causal validation results for the 11 identified proteins in CAS.

Protein	UniProt	OR (95% CI)	*P_discovery_*	*P_replication_* ^a^	*P_reverse_*	Colocalization ^b^ PPH_4_	Panel	Tier
PCSK9	Q8NBP7	1.28 (1.22–1.34)	1.61 × 10^−25^	5.64 × 10^−5^	0.64	0.999/0.999	Cardiometabolic	1
CELSR2	Q9HCU4	0.82 (0.79–0.86)	3.88 × 10^−21^	1.70 × 10^−29^	0.04	0.997/0.994	Inflammation	1
APOE	P02649	0.94 (0.92–0.95)	2.70 × 10^−11^	1.44 × 10^−21^	0.95	0.000/0.000	Inflammation	2
LPA	P08519	1.14 (1.09–1.2)	5.51 × 10^−9^	4.11 × 10^−90^	0.45	0.000/0.000	Inflammation	2
IL6R	P08887	0.96 (0.95–0.98)	7.77 × 10^−8^	2.05 × 10^−5^	0.78	0.986/0.972	Cardiometabolic	1
FN1	P02751	0.9 (0.86–0.94)	3.22 × 10^−7^	3.47 × 10^−8^	0.53	0.972/0.945	Inflammation	1
APOA5	Q6Q788	1.06 (1.03–1.09)	2.17 × 10^−6^	1.62 × 10^−4^	0.44	0.000/0.000	Cardiometabolic	2
AGER	Q15109	0.91 (0.87–0.94)	2.58 × 10^−6^	0.12	0.64	0.020/0.009	Inflammation	3
CD4	P01730	0.9 (0.86–0.94)	5.33 × 10^−6^	0.71	0.12	0.983/0.966	Inflammation	3
TGFB1	P01137	1.16 (1.09–1.23)	5.56 × 10^−6^	2.77 × 10^−6^	0.62	0.450/0.290	Inflammation	2
SPARCL1	Q14515	0.94 (0.91–0.97)	1.05 × 10^−5^	5.08 × 10^−3^	0.37	0.963/0.929	Cardiometabolic	1

^a^ Replication validation results from UK Biobank and GWAS Catalog, displaying only the most significant findings. ^b^ Prior probability p12 (1 × 10^−5^/5 × 10^−6^).

## Data Availability

The results and conclusions of this study are available in the main text and Supplementary Materials. The detailed results of the MR, SMR, colocalization analysis, and reverse MR are provided in the Supplementary Tables. The statistical analysis methods, quality control procedures for pQTL data and GWAS phenotype data, and other specific details can be accessed on the source website. The atherosclerosis GWAS phenotype data were obtained from the Pan-UK Biobank (https://pan.ukbb.broadinstitute.org/docs/summary, accessed on 20 January 2025), the FinnGen cohort (https://finngen.gitbook.io/documentation/r10, accessed on 15 December 2024), and the GWAS Catalog (GCST90043957 and GCST90436075). The inflammation- and cardiovascular-related pQTL data were obtained from the UKB-PPP (https://metabolomips.org/ukbbpgwas/, accessed on 20 January 2025) and Iceland cohorts (https://www.decode.com/summarydata, accessed on 15 January 2025). The single-cell RNA-seq data were obtained from the Gene Expression Omnibus (GEO) database (GSE252243). The single-cell expression matrix is accessible via the GEO database (GSE252243).

## Supplementary Materials

The following supporting information can be downloaded at: https://www.mdpi.com/article/10.3390/ijms26136201/s1.

## Abbreviations

CAS, coronary atherosclerosis; MR, Mendelian randomization; pQTL, protein quantitative trait loci; SMR, summary-data-based Mendelian randomization; PPI, protein–protein interaction; CAD, coronary artery disease; MI, myocardial infarction; HF, heart failure; CHD, coronary heart disease; AIT, adaptive intimal thickening; SMCs, smooth muscle cells.
