# Supplementary material for: Identification of Potential Therapeutic Targets for Coronary Atherosclerosis from an Inflammatory Perspective Through Integrated Proteomics and Single-Cell Omics
Source: Int J Mol Sci. 2025 Jun 27;26(13):6201. doi: 10.3390/ijms26136201 (PMC12250334; doi:10.3390/ijms26136201)
Supplement: Supplementary file 1 [file ijms-26-06201-s001.zip › Supplementary Figure.pdf]

Supplementary Figures

Supplementary Figure 1. The association plot of the colocalization region between PCSK9 and CAS.

Supplementary Figure 2. The association plot of the colocalization region between CELSR2 and CAS.

Supplementary Figure 3. The association plot of the colocalization region between APOE and CAS.

Supplementary Figure 4. The association plot of the colocalization region between LPA and CAS.

Supplementary Figure 5. The association plot of the colocalization region between IL6R and CAS.

Supplementary Figure 6. The association plot of the colocalization region between FN1 and CAS.

Supplementary Figure 7. The association plot of the colocalization region between APOA5 and CAS.

Supplementary Figure 8. The association plot of the colocalization region between CD4 and CAS.

Supplementary Figure 9. The association plot of the colocalization region between SPARCL1 and CAS.

Supplementary Figure 10. The association plot of the colocalization region between AGER and CAS.

Supplementary Figure 11. The association plot of the colocalization region between TGFB1 and CAS.

Supplementary Figure 12. Interactions between CAS drug targets and identified potential drug targets (PCSK9, IL6R, CELSR2, FN1, SPARCL1, APOE, LPA, APOA5, TGFB1, and AGER).

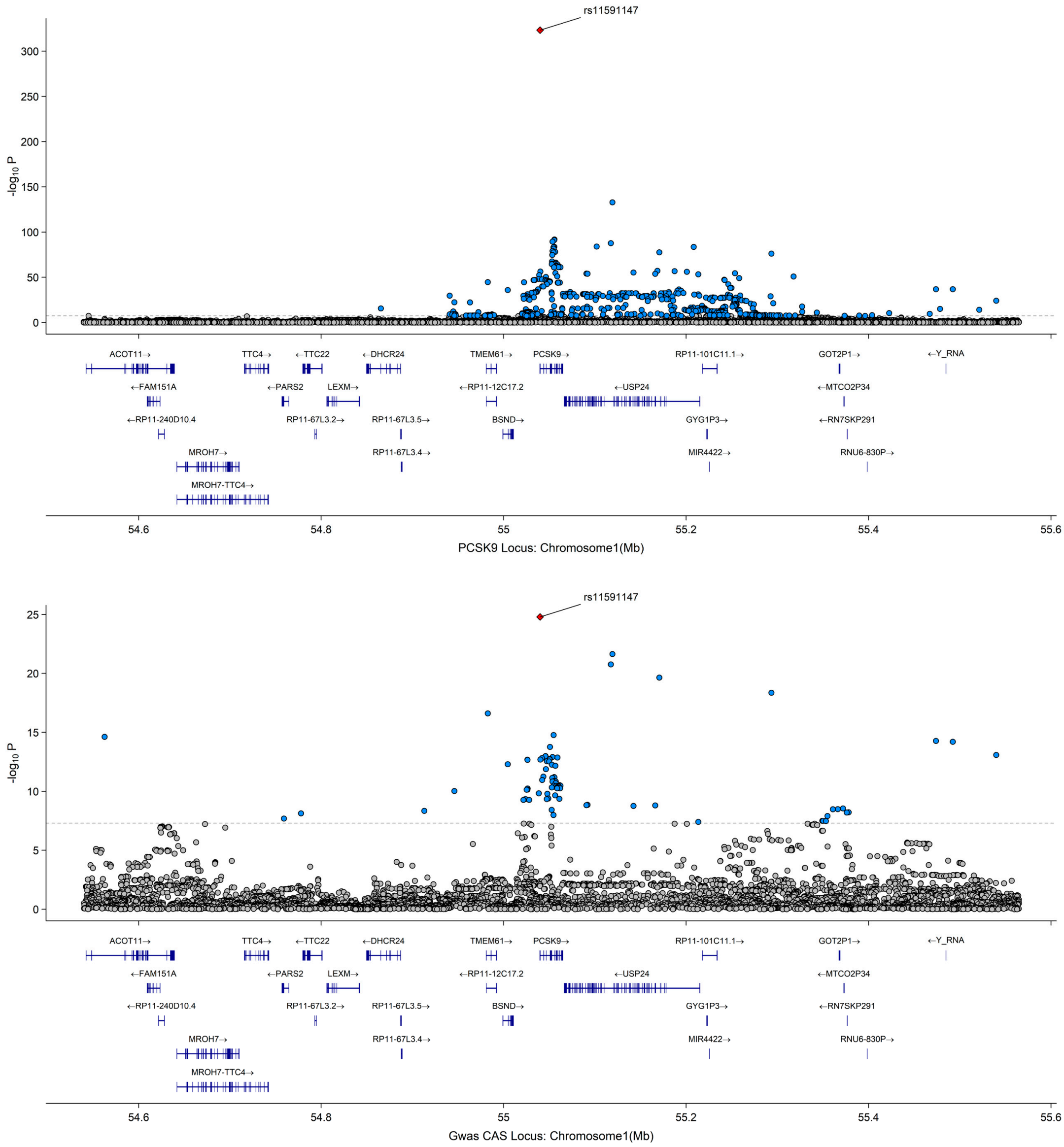

Supplementary Figure 1. The association plot of the colocalization region between PCSK9 and CAS.

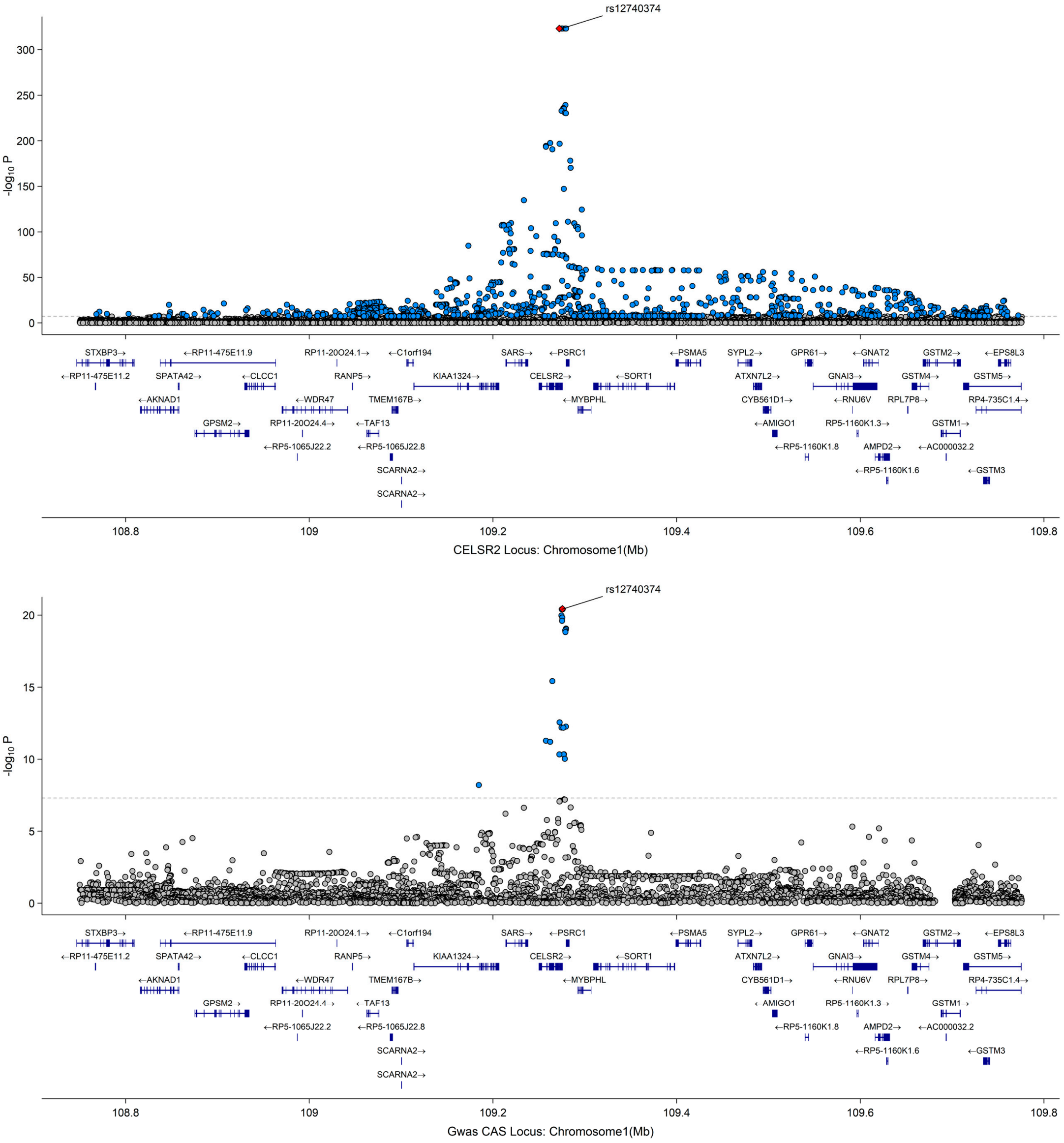

Supplementary Figure 2. The association plot of the colocalization region between CELSR2 and CAS.

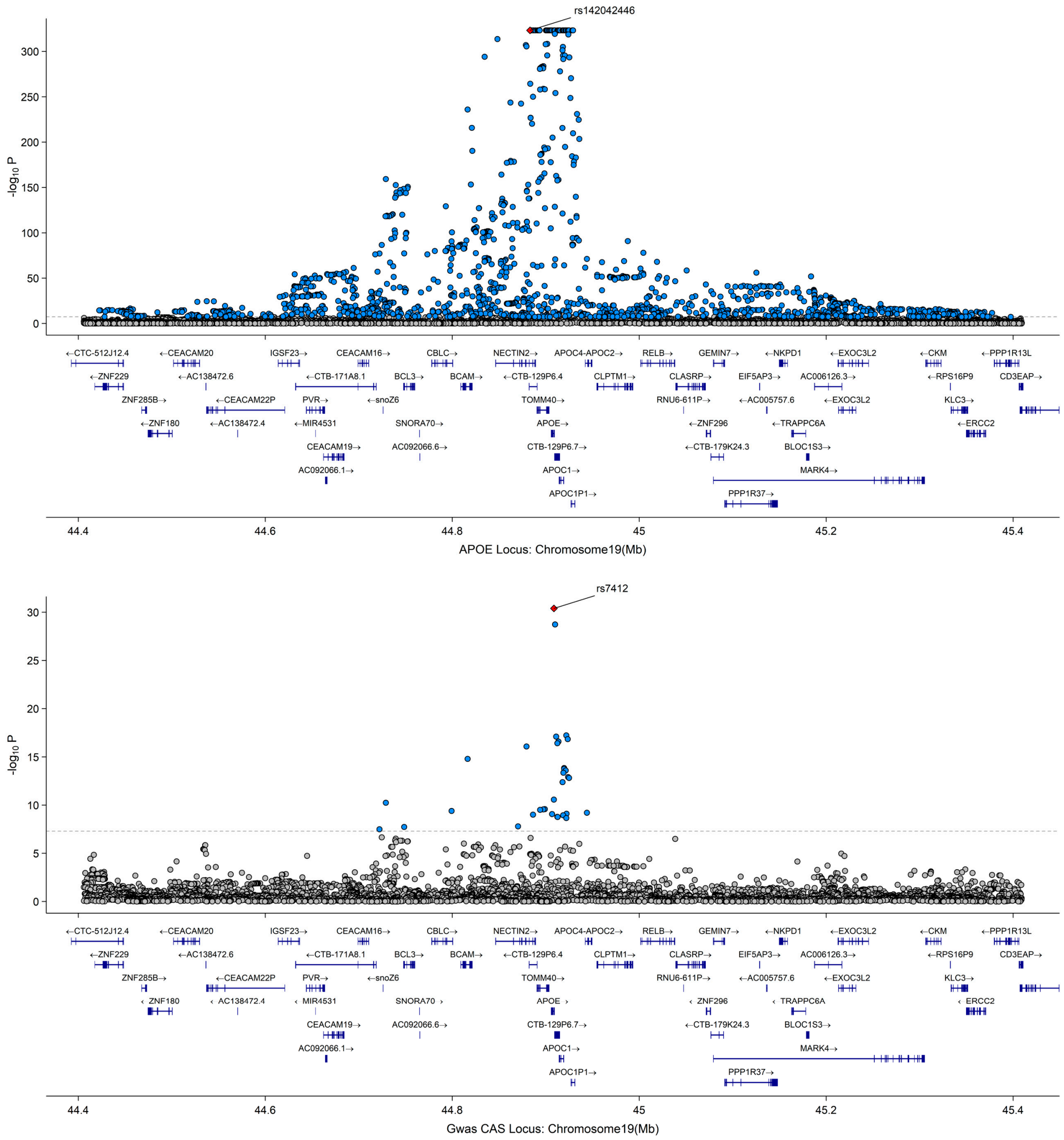

Supplementary Figure 3. The association plot of the colocalization region between APOE and CAS.

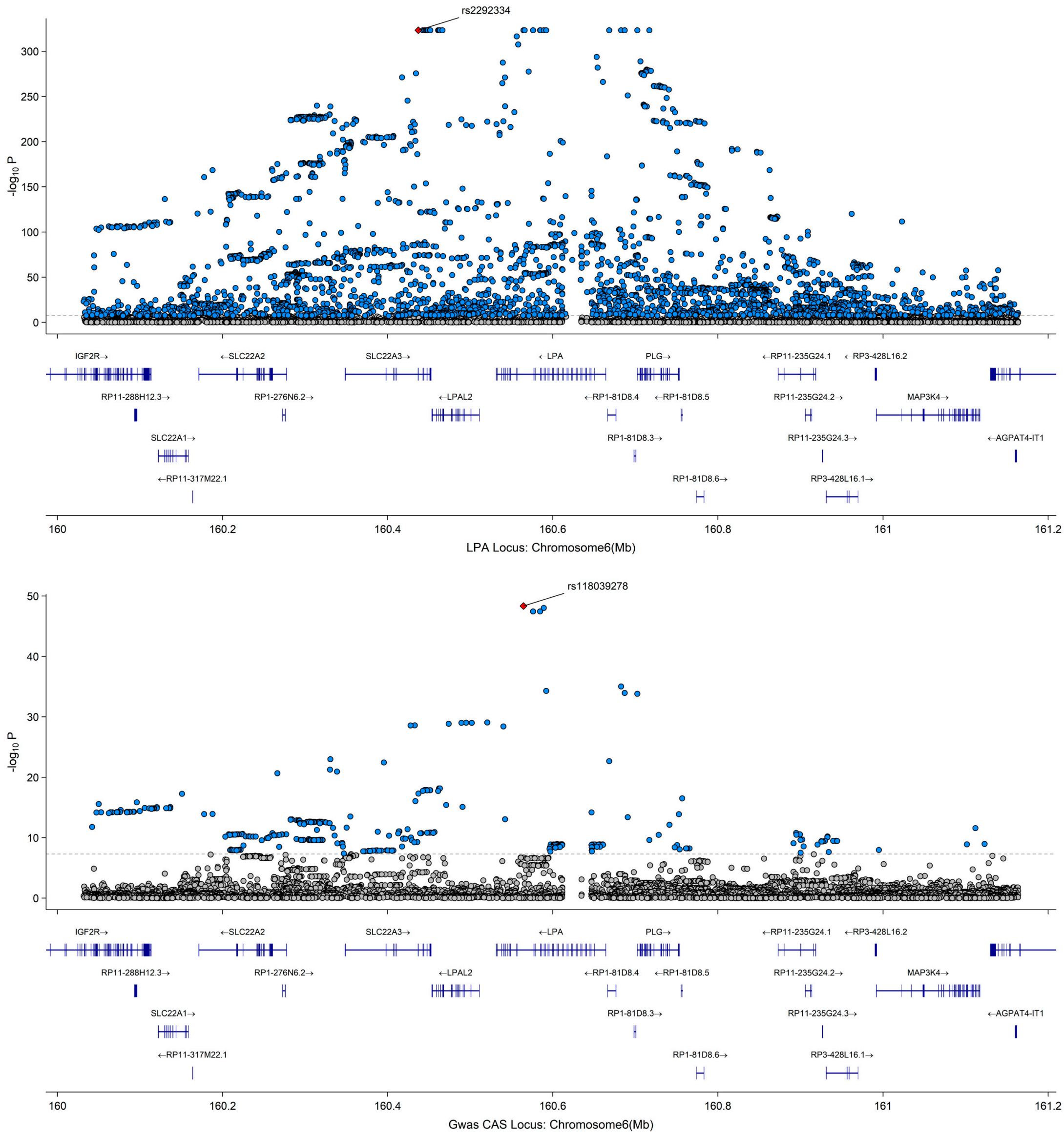

Supplementary Figure 4. The association plot of the colocalization region between LPA and CAS.

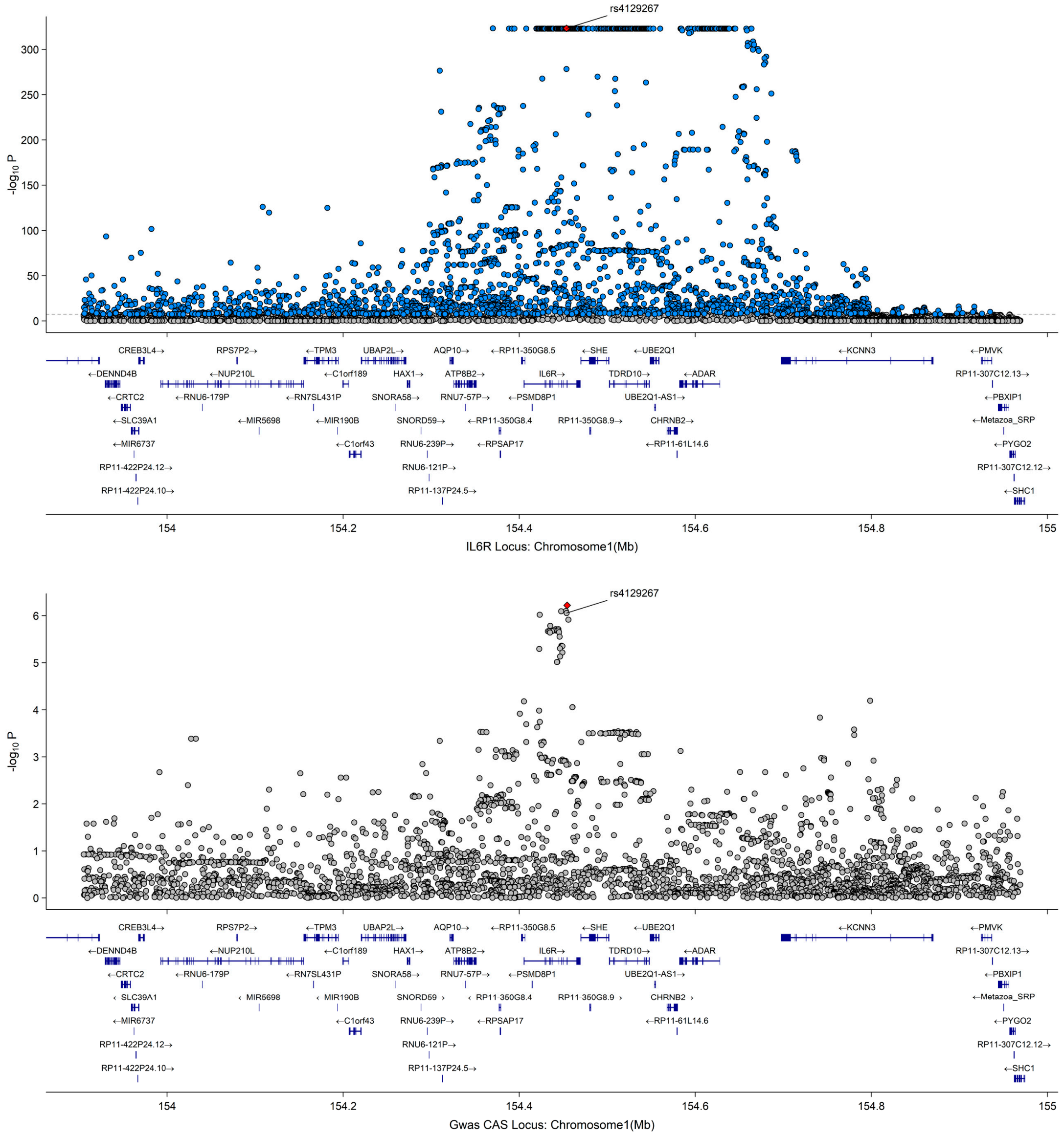

Supplementary Figure 5. The association plot of the colocalization region between IL6R and CAS.

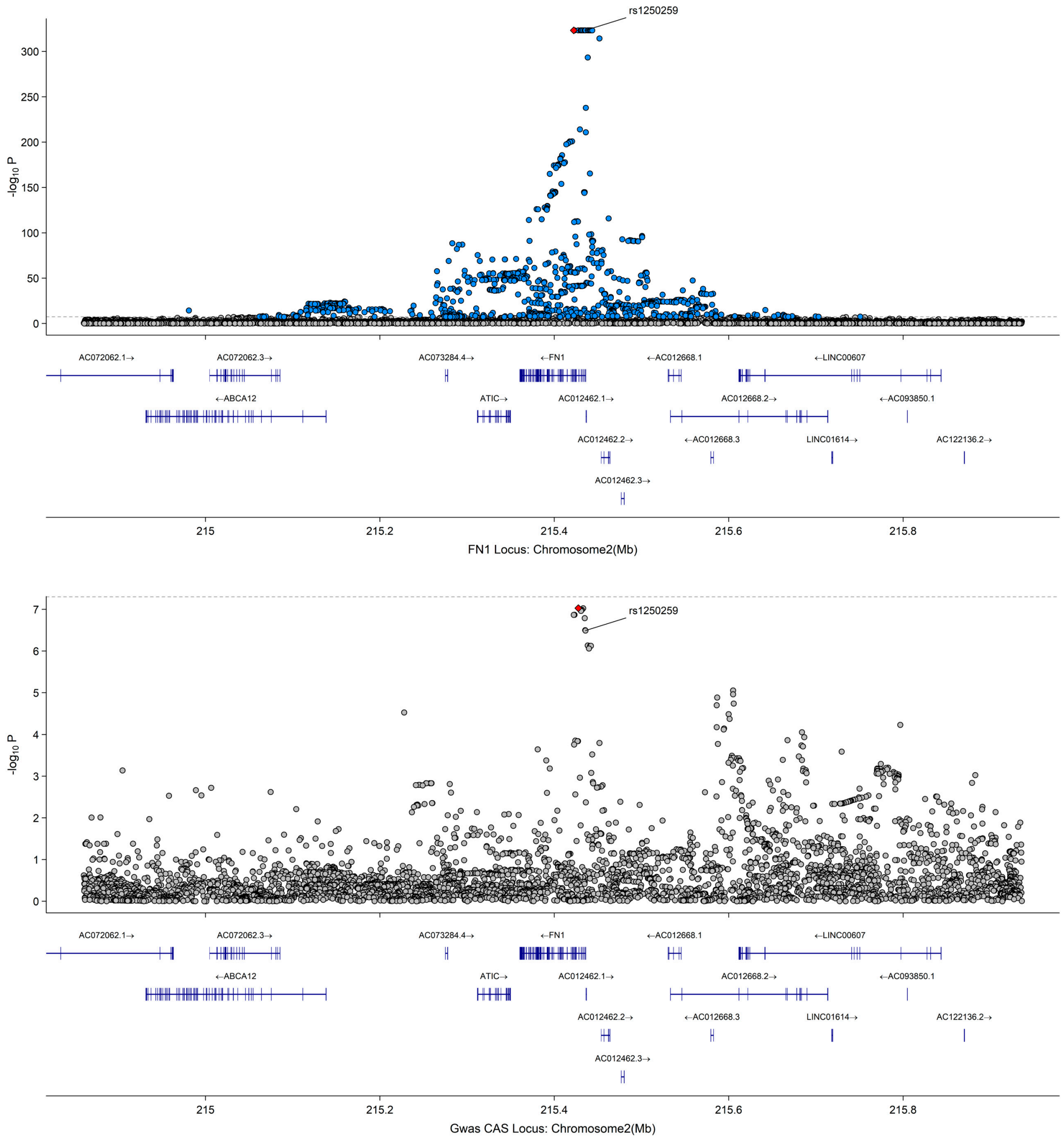

Supplementary Figure 6. The association plot of the colocalization region between FN1 and CAS.

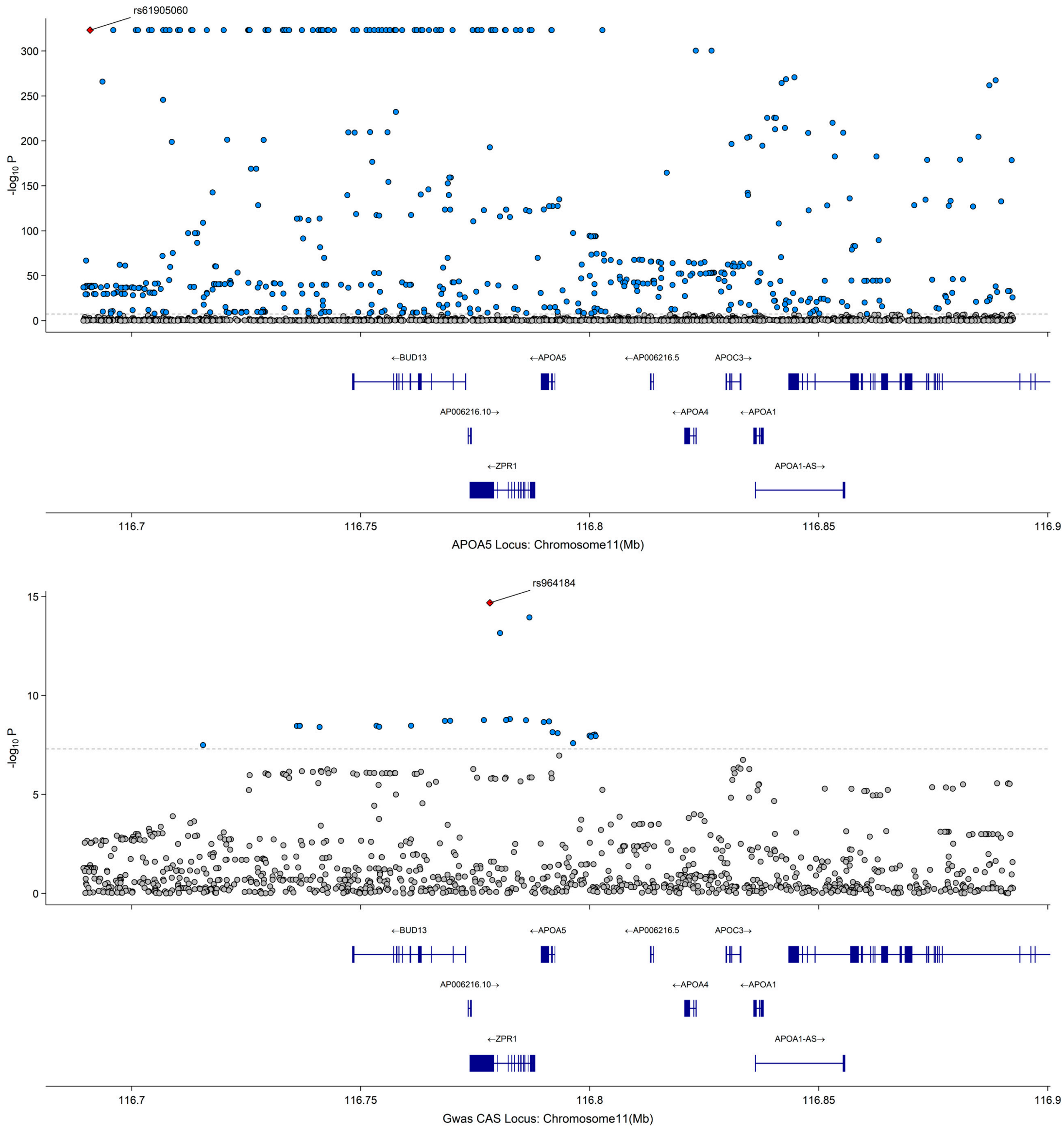

Supplementary Figure 7. The association plot of the colocalization region between APOA5 and CAS.

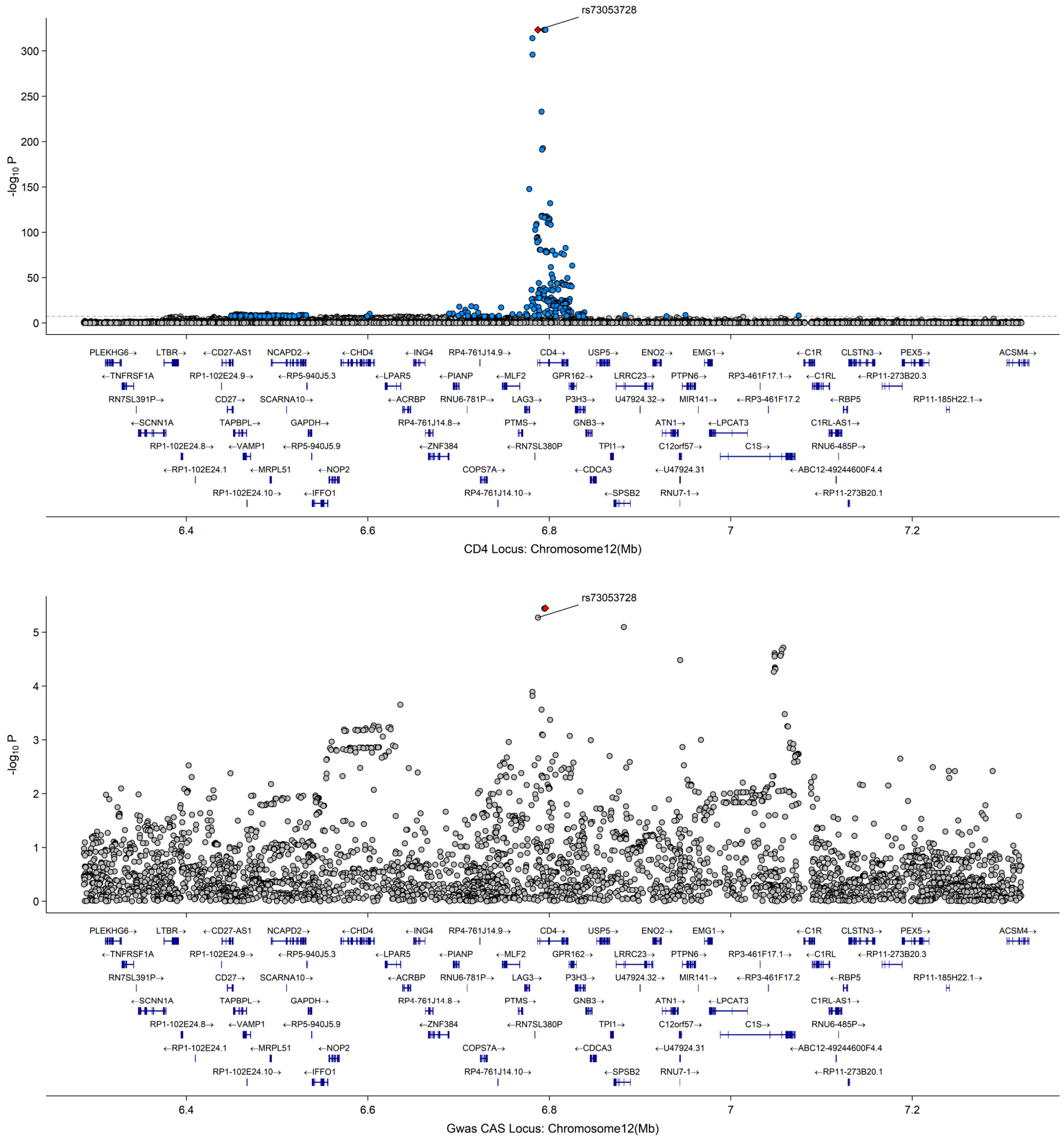

Supplementary Figure 8. The association plot of the colocalization region between CD4 and CAS.

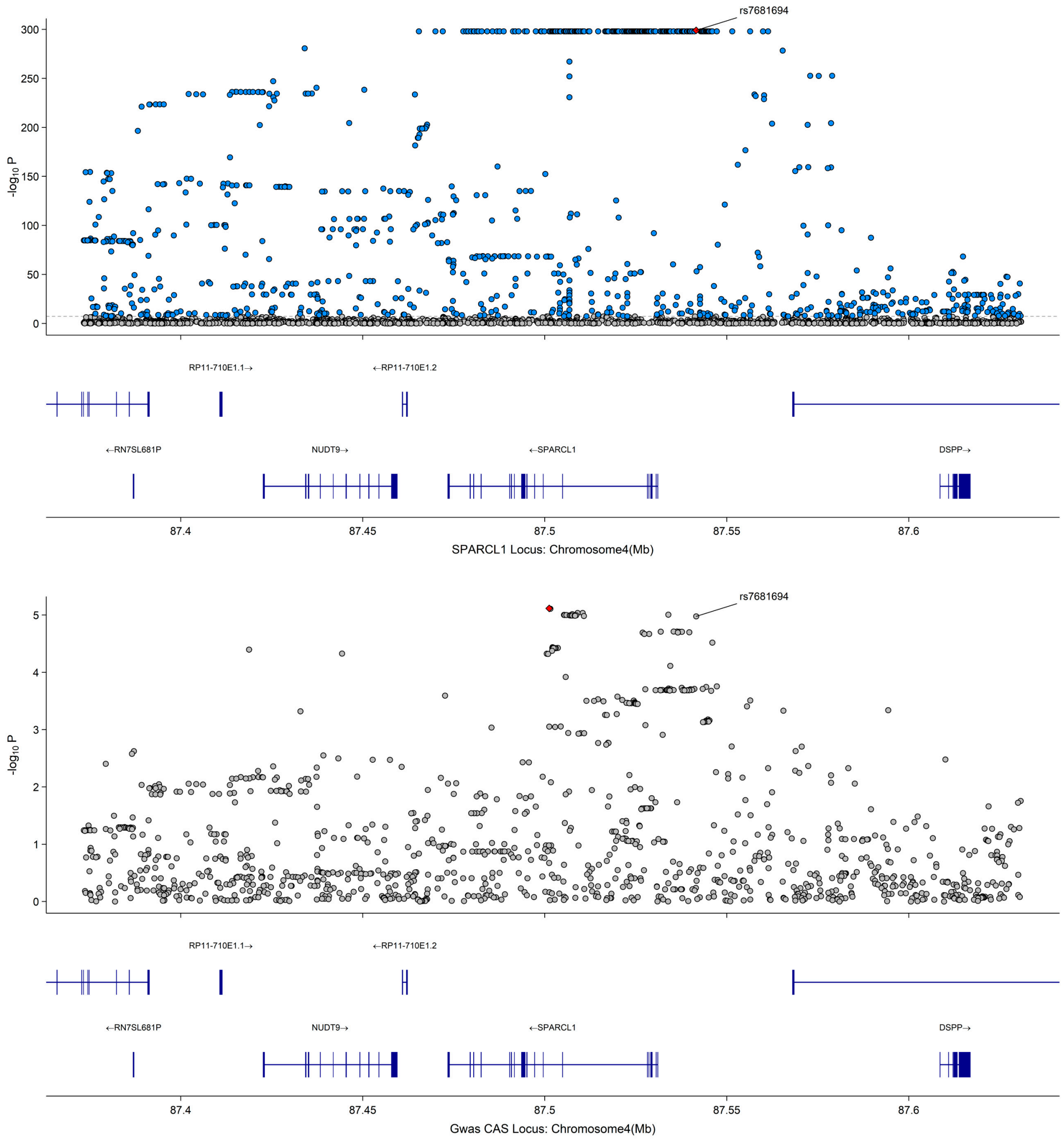

Supplementary Figure 9. The association plot of the colocalization region between SPARCL1 and CAS.

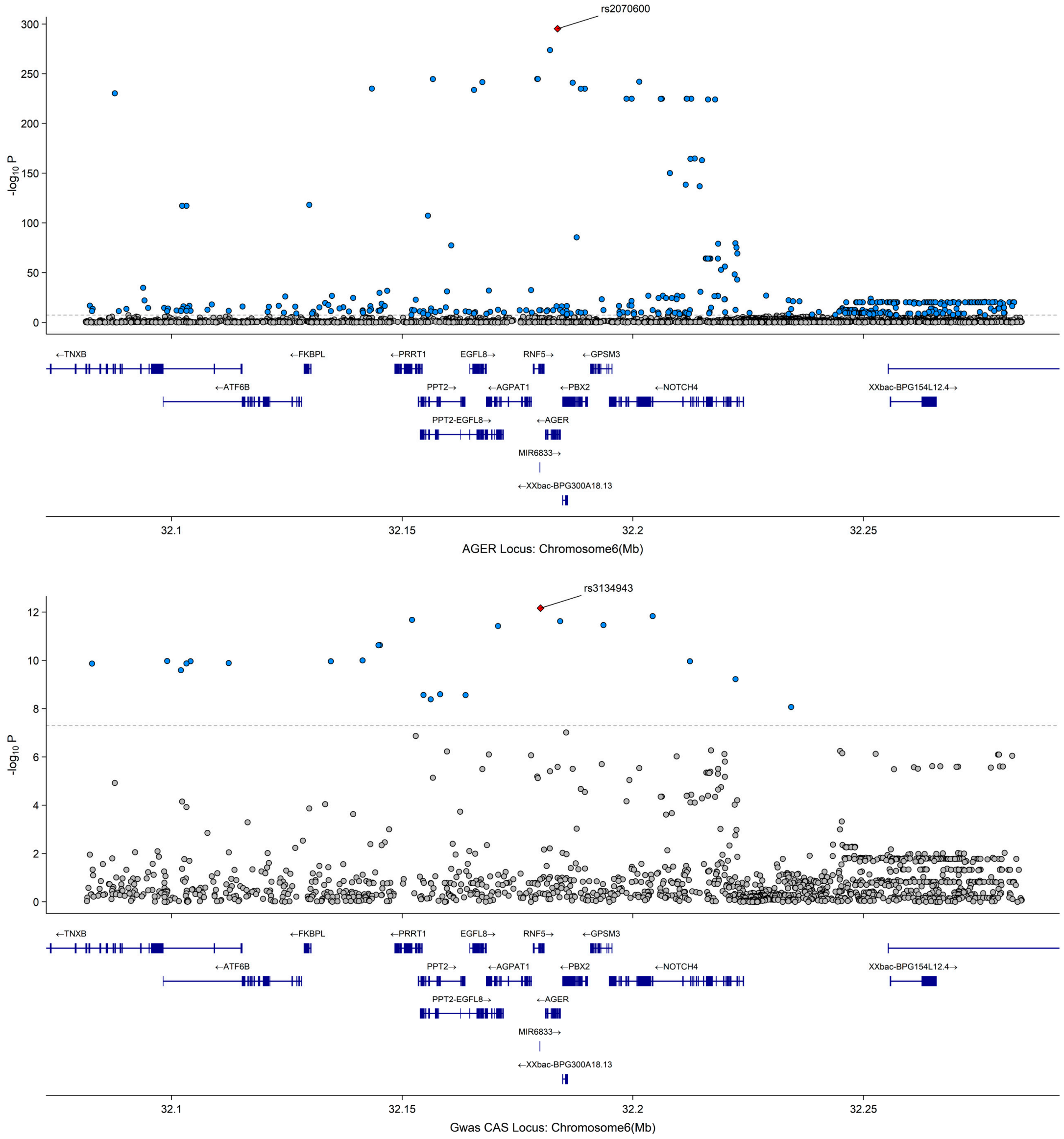

Supplementary Figure 10. The association plot of the colocalization region between AGER and CAS.

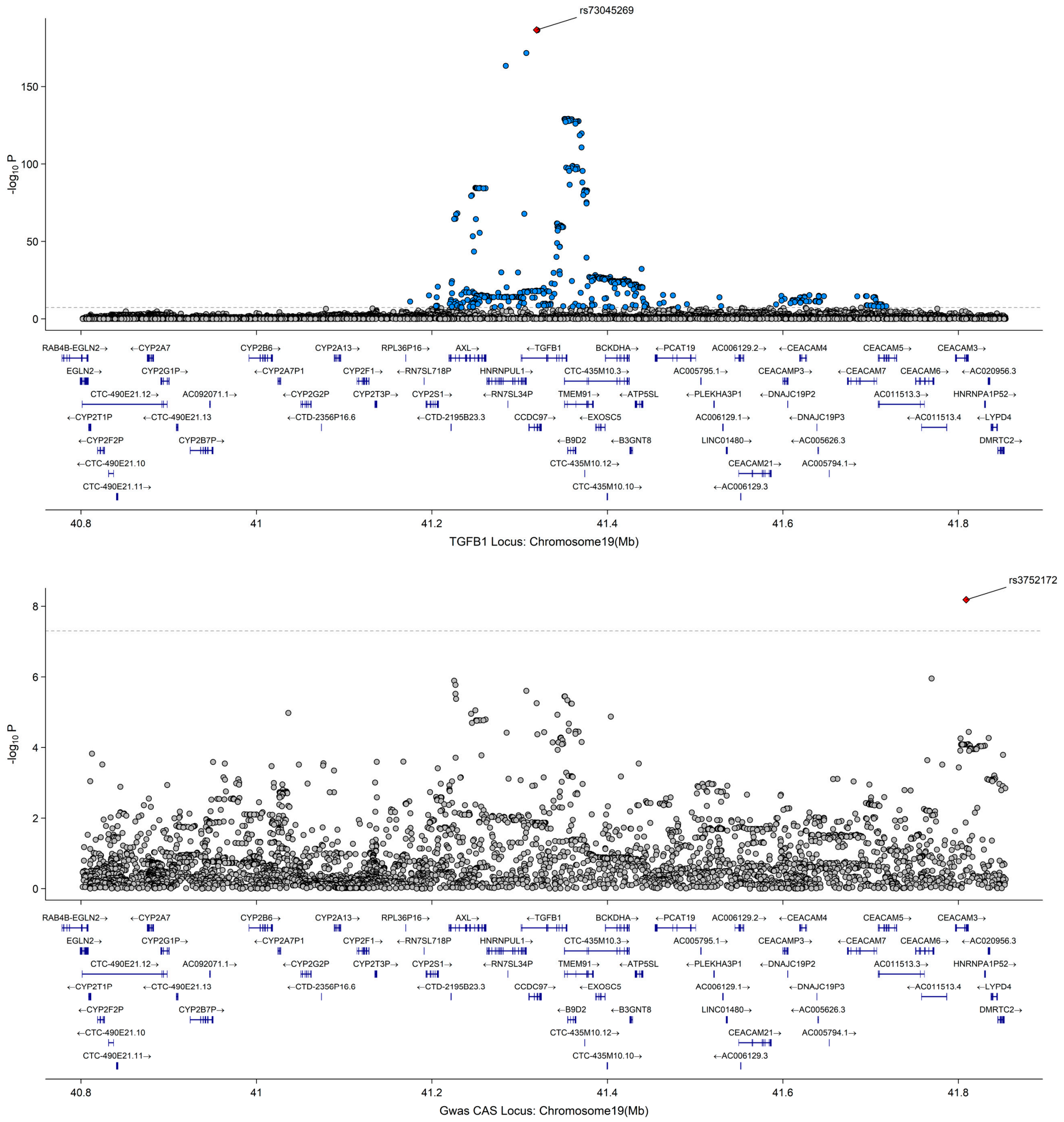

Supplementary Figure 11. The association plot of the colocalization region between TGFB1 and CAS.

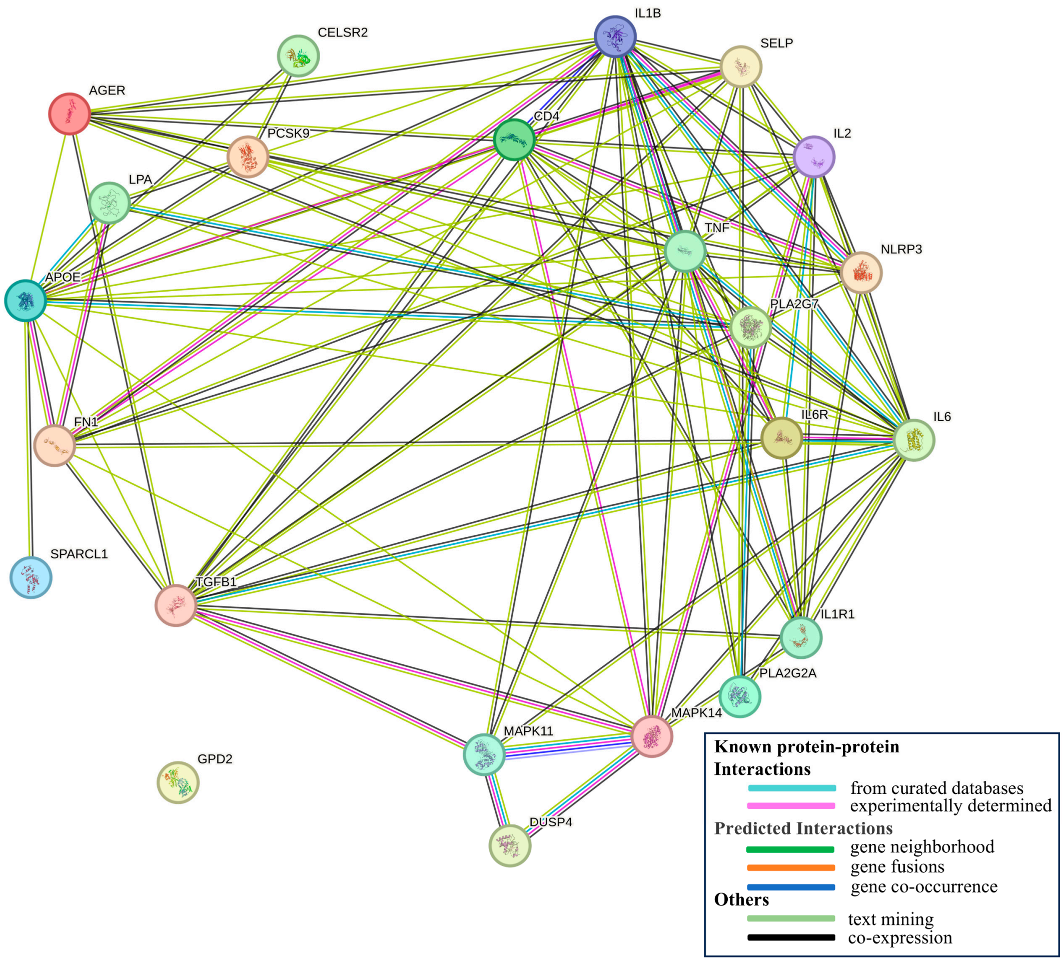

Supplementary Figure 12. Interactions between CAS drug targets and identified potential drug targets (PCSK9, IL6R, CELSR2, FN1, SPARCL1, APOE, LPA, APOA5, TGFB1, and AGER).
